# Meeting of the Royal Sanitary Institute in Bristol

**Published:** 1905-04-15

**Authors:** 


### MEETING OF THE ROYAL SANITARY INSTITUTE IN BRISTOL.

### DISCUSSION ON ISOLATION HOSPITALS.

On Saturday, April 8th, a sessional meeting of the Royal Sanitary Institute was held in Bristol. The West of England and South Wales Branch of the Incorporated Society of Medical Officers of Health joined forces with the Institute, and the visitors, to the number of 80, were received at 11 o'clock in the Council Chamber, by the Right Hon. the Lord Mayor of Bristol, who cordially welcomed the Institute to Bristol, touched on its past history and on the continuous and useful efforts it had put forth over a series of years for the improvement of sanitation in England, and concluded by wishing the members a pleasant visit to the city, and pleasant memories to take away.

An adjournment was then made to the Civil Court, Guildhall, where the formal business of the day began. The chair was taken by the Chairman of the Council of the Institute, Mr. Whittaker, B.A., F.R.S., F.G.S. After the Secretary, Mr. White Wallis, had read the minutes of the previous meeting the Chairman called on Dr. D. S. Davies, Medical Officer of Health for the City of Bristol and General Medical Superintendent of the City Hospitals, to open a discussion on isolation hospitals.

Dr. Davies explained that he wished to approach the consideration of the question from a somewhat novel standpoint, and, instead of appealing to statistics, either of different towns or of the same town at different periods, he would prefer to consider the theory of hospital isolation and the essential conditions upon which its success as a preventive measure was based.

It seemed to be evident, upon consideration, that in order to secure the success of isolation the four following "essential conditions" must be fulfilled.

1. The patient has not handed on his infection to any other person before his seclusion commenced.
2. The patient has left behind no infected material, or, if he has, such infected material must be rendered sterile.
3. The patient is kept perfectly secluded through the whole of his illness and convalescence from direct or indirect communication of his disease to others.
4. The patient is perfectly recovered before he is cleansed and discharged from seclusion.

If these conditions are fulfilled, isolation succeeds in preventing the spread of disease, if any one of them fails, isolation does not succeed, but failure from non-fulfilment of the essential conditions of success does not affect the principle of isolation.

The difficulty in securing the fulfilment of these essential conditions varies with the natural history of the disease. Small-pox is one of the most straightforward of diseases, and exhibits in fullest degree the value of isolation, especially when this is ready beforehand for first introductions. The reason for this is that the organism of small-pox is so highly organised that it is "selective" as to soil; it cannot grow on unsuitable soil, it dies out on exhausted soil; contacts can be rendered unsuitable by inducing vaccinia. Hence, in this disease, a patient may contract it and suffer from it, but, upon recovery, the organism can no longer persist in the patient, and his recovery is perfect: therefore, "return cases" are unknown. Again, the disease, if not able to attack a contact, fails entirely to grow upon his tissues, so we get no "carrier" cases. Finally, owing to this selective character of small-pox, it tends to disappear utterly from communities, so that upon reintroduction of the disease, we start upon equal terms, and have no endemic prevalence to confuse issues, but have only to deal with the spread in geometric increase from each introduced centre. For ten years Bristol, in common with many other towns, had been uniformly successful in limiting the many introductions of this disease, in which hospital isolation had been one most necessary and effective factor.

The speaker contrasted the behaviour of diphtheria, a disease in which the causal organism was not selective—would grow upon almost any soil, in a variety of situations—throat, nose, ear, or upon abrasions—and could produce not only definite clinical cases, but also "carrier cases," in which a person, apparently well, carries about virulent organisms upon his tissues and constitutes a standing source of danger to other susceptible persons. Hence arose innumerable opportunities for "missed" cases, which mixed freely with others and kept up the infection. Furthermore, apparent recovery by no means necessarily involved cessation of the growth of the organism, which might persist for long periods, and, gaining access to deep recesses of the nasal cavity, to the frontal sinuses, or to the antrum, might lead to the development of chronic cases. A disease of this nature led to the establishment of an endemic condition during epidemic invasion and confused the value of hospital isolation in dealing with the disease. The necessity for hospitals in dealing with urgent cases requiring operation, and in securing the due and efficient use of antitoxin in treatment, remained most evident, although their use in absolute prevention was far less evident and less effective than in the case of small-pox.

Scarlet fever was next dealt with as the disease in regard to which the value of hospital isolation had been most seriously questioned.

If the four "essential conditions" could be secured there was no doubt that isolation in the case of scarlet fever would be as effectual as in the case of small-pox: but this was difficult to secure because in scarlet fever there was considerable tendency to the development of a chronic condition in some instances, leading to "return cases," and a still greater trouble of late years was the occurrence of an extremely mild variety of the disease which obscured the symptoms, led to non-recognition, and involved a large number of "missed" cases. The mildness of the variety, though it led to a low case-mortality, was therefore not entirely favourable, and undoubtedly conduced to widespread extension.

The speaker commented on the difficulty of obtaining any really reliable indications by comparing the statistical records of one town with those of another; all the factors involved were variable, and useful comparison was impossible. The success of isolation is intimately bound up with, and can only justly be considered in relation to, the fulfilment of the four "essential conditions" of success.

The importance of "anticipatory" provision of isolation accommodation, so as to deal with first cases and their developments, rather than with a fully-developed epidemic, was insisted upon; and the necessary amount of provision was discussed.

The isolation accommodation at present available for the city of Bristol amounts to 190 beds, part of which is in temporary wards. The full accommodation should equal 358 beds. The fever beds available at Ham Green now number 135.

In conclusion the speaker submitted to the meeting "That opposition to hospital isolation arose from lack of appreciation of the essential conditions for success; and that hospital isolation provision was imperative for centres of population, and the proportion of one bed per 1,000 population was a good working minimum."

After discussion, this proposition was seconded by Dr. Howard Jones and was carried unanimously.

Mr. T. H. Yabbicom, M.Inst.C.E., City Engineer of Bristol, followed with "A Short Description of the Isolation Hospitals Provided by the Corporation of Bristol."

He pointed out that the area of the city was now 17,004 acres, and the population was over 358,000. Hospitals had been established at two sites: at Novers Hill for small-pox, and at Ham Green for fever cases. The greatest length of the city is 9½ miles, and its breadth nearly 7 miles. The Novers Hill site contains an administration building with day-rooms and 12 bedrooms, an observation block for eight beds, and three temporary wooden blocks containing 35 beds; a laundry and porter's lodge are also provided. The site covers 13 acres, so that there is ample room for extension.

The Ham Green site contains 99 acres, of which 38 have been devoted to hospital purposes. It is 4 miles from the centre of the city, on a hill 80 feet above high water, on the south bank of the river Avon.

The geological formation is dolomitic conglomerate. Borings have failed to yield water, so this is obtained from the Portishead Water Company. The administration block is adapted from the existing mansion, with recent additions. The laundry contains a complete steam plant; the electric light is produced on the premises; disinfecting apparatus and stabling are provided. The hospital buildings now consist of seven blocks, substantially built in late Gothic style, in red brick with freestone dressings, and slated roofs. Two observation blocks are provided, and five ward pavilions, spaced 60 feet apart; four of these are one-storey buildings for 17 patients in each. The new block is a two-storey building. Opening out of the central hall on the ground floor are two wards, each 72 feet long by 26 feet wide, for 12 beds each, with a cubic allowance of 2,028 feet. On the first floor are two similar wards. A small ward is provided on each floor. The height of the wards is 13 feet. Double-sash windows, the top portion of which fall inwards, afford light and ventilation. Two of Shorland's ventilating open double fire grates are in the centre of each ward; and there are also low-pressure hot water radiators round the walls. The sewage is chemically treated before being discharged into the Avon.

The discussion was opened by Dr. Colston Wintle, Chairman of the Bristol Health Committee, who described the steps taken in the city of Bristol to provide isolation for suitable cases of phthisis in the early stages when permanent benefit was likely to result. The Corporation of Bristol had secured 20 beds, which they maintained, at the Winsley Sanatorium for Consumption near Limpley Stoke. Some years ago it would have raised astonishment to attempt to deal with consumption as a curable disease or a preventable disease, but now it was recognised that much good might be effected in this direction, and he thought Bristol had made an excellent move. There was considerable demand for the beds, and the reports of the cases already admitted was encouraging. The speaker quoted statistics showing that, of early suitable cases sent in for sanatorium treatment, it might be expected that 95 per cent. would be discharged perfectly fit for work, while of the bad cases, some 40 per cent. would probably be so much improved as to be able to resume work.

Dr. Herbert Jones, Medical Officer of Health for the Herefordshire Combined District, supported Dr. Davies' contention that statistics were inconclusive as to the value of isolation hospitals, but that experience fully demonstrated their usefulness. Districts were not always comparable. The large house might improvise a fever ward in a spare room, but the isolation hospital was necessary as the "poor man's spare room."

The great cost of hospitals was the bar to their extension, and, so far as the speaker could see, no help was to be expected either from the Local Government Board or from the architects, who, no doubt, erected substantial and elegant buildings but paid too little attention to standard sizes, so that linoleum cut to waste, blind widths were difficult to adapt to windows, and so forth, all small matters perhaps in themselves, but adding to the general cost. The economy of fitting lavatory basins in nurses' bedrooms to the saving of crockery was insisted on. The speaker questioned whether a two-storey building was, in reality, any great saving on a one-storey building.

Dr. Howard Jones, medical officer of health, Newport, Mon., said that in Newport their accommodation just met the standard—1 bed per 1,000 inhabitants—and just proved sufficient for their needs. He entirely agreed with the writer of the paper that the mildness of an epidemic, leading to much risk of "missed" cases, conduced to the wide spread of the disease. The utility of hospitals seemed to him to be indisputable; especially were they valuable in diphtheria. The speaker advocated the free use of antitoxin early in cases, and its injection into contacts as a prophylactic. Full doses of antitoxin were advisable—a fact not yet appreciated by the War Office, who recently censured a medical officer, who requisitioned 12,000 units, for extravagance, pointing out that 2,000 to 4,000 units would have been ample. (Laughter.)

Dr. Martin, medical officer of health for the county of Gloucester, endorsed the necessity for securing the "four essential conditions," emphasised the importance of "missed" cases in promoting the spread of disease, remarked on the varying cost of hospitals—from £160 to £500 per bed—and suggested that architects should co-ordinate and arrive at a working standard of economy combined with efficiency. The speaker pointed out that the cost of small-pox hospitals would be entirely avoided if the English Government would insist upon vaccination and re-vaccination, and follow the example of Germany, where, as Dr. Bruce Low's recent report to the Local Government Board showed, an empty ward in a general hospital was sufficient for the few cases of small-pox, generally introduced from neighbouring countries less well protected, which still require treatment in Germany.

Mr. Reed, Surveyor to the City of Gloucester, criticised some points in Mr. Yabbicom's paper; he agreed as to the unsatisfactory nature of a converted building, instancing a case where, in patching up an old house, it was found necessary first to raise the ceiling to give head room on the ground floor, then to raise the first-floor ceiling, and finally to raise the roof. (Laughter.) He commended the use of Shorland's ventilating stoves, as the supply of fresh warmed air was duly provided for, instead of being left to chance.

Dr. Tubb Thomas, Medical Officer of Health for the County of Wilts, said that, if the large city of Bristol was inadequately provided for, the county districts could not well be blamed. In Wiltshire he was glad to be able to state that good progress had been made in this direction, and already satisfactory provision was available for two-thirds of the districts in the county. The cost was at present excessive, and the question of possible reduced cost was being seriously taken up. The speaker believed that, if sufficient trouble were taken, scarlet-fever could be dealt with as efficiently by isolation as small-pox, and instanced a large watering-place where he knew this had been done. The local practitioners knew that, if the fever was allowed to spread, their visitors would not come, and they would have no harvest; so they worked hard in their own interest and succeeded. (Laughter.) In rural districts the hospital was generally inadequately staffed, a caretaker only in charge, and nurses had to be sent for when it was commissioned. A lay authority naturally said, "Oh, don't let us open the hospital for one case," so they waited until there was an epidemic, the hospital naturally failed to stop it and got undeserved opprobrium. The Wiltshire County Council were constructing isolation hospitals at a cost not exceeding £300 per bed.

Dr. Fletcher, resident medical officer at Ham Green Hospital, pointed out that while it was comparatively simple to determine convalescence in chicken-pox, measles, or small-pox, it was in many cases of scarlet fever or diphtheria most difficult or impossible. Convalescent wards were really necessary to entirely guard against return cases. He considered that discovery of the causal organism of scarlet fever, and its recognition by easily applied methods, would render much help in dealing with this disease. No diphtheria cases were discharged without a double bacterial test, at an interval, and return cases, although not unknown, were very rare. In his experience mild forms of diphtheria were the ones from which chronic persistence was to be most feared; bad cases excited more constitutional reaction, and the cases cleared up more perfectly. He believed in the administration of antitoxin in free doses, even up to 24,000 or 30,000 units. It should be given at the earliest possible moment; if given early, the patient only needs tiding over 24 or 36 hours; intubation is preferable to tracheotomy, and in that case, even if intubation fails, tracheotomy can be utilised as a last resort. Cases received late into hospital often show the ill-effects of home treatment and neglect—mastoid abscess, kidney mischief, and other troubles generally avoided in hospitals, which he considered indispensable.

Dr. Heaven, medical officer of health for Keynsham, emphasised the value of hospitals in rural districts, where also their use can be more readily demonstrated than in large towns. He questioned if the proportion of one bed per 1,000 population is always sufficient, and stated that his experience of a joint hospital board was not encouraging. "Missed" cases were undoubtedly fertile sources of the spread of disease in large centres of population.

### Visit to Ham Green Hospital.

In the afternoon, 60 of the visitors availed themselves of the invitation of the Health Committee to visit Ham Green Hospital. Brakes were provided, and the weather proved extremely favourable. On arriving at the hospital the visitors were welcomed by Dr. Wintle, chairman of the Health Committee, and then visited and closely inspected the new blocks under the guidance of the city engineer and the general medical superintendent, and examined the laundry, electric lighting station, and disinfecting plant. After this Dr. Wintle entertained the company at tea, and they shortly afterwards drove back to the City, expressing themselves highly pleased with his kind hospitality, and having thoroughly enjoyed their visit to the hospital, the charming situation of which was much admired.

